# Individualised network connectivity-based targeting to improve outcomes of transcranial magnetic stimulation in difficult-to-treat depression

**DOI:** 10.1192/j.eurpsy.2025.1973

**Published:** 2025-08-26

**Authors:** T. T. Raij, E. Komulainen, D. B. Aydogan, M. Stenroos

**Affiliations:** 1 University of Helsinki and Helsinki University Hospital, Helsinki; 2 University of Eastern Finland, Kuopio; 3 Aalto University School of Science, Espoo, Finland

## Abstract

**Introduction:**

Transcranial magnetic stimulation (TMS) is an established treatment for difficult-to-treat depression (DTD), demonstrating moderate efficacy. In comparison to other non-invasive treatments in psychiatry, TMS can be relatively accurately directed at specific cortical subregions. TMS is typically targeted at the dorsolateral prefrontal cortex (DLPFC) based on scalp measurements. However, the DLPFC is a large and functionally heterogeneous region, with inter-individual variability in the localisation of target functions. The functional connectivity of the stimulation target with other brain areas has been shown to correlate with treatment outcomes. Nonetheless, the clinical benefits of connectivity-based individualised targeting remain to be demonstrated in randomised clinical trials, and the connections to be considered in such targeting are still inadequately understood.

**Objectives:**

To test whether individualised network connectivity-based localisation improves outcomes, and to further investigate the relationship between clinical results and the stimulated connections

**Methods:**

In this ongoing study, we acquire functional and structural MRIs from adult volunteers with DTD, referred to clinical TMS. We focus on brain regions associated with depression and emotion regulation to target the core affective symptoms of depression. We compute the individual connectivity distribution of these regions within the DLPFC. We then employ a realistic individual head conductor model to determine the coil position that aligns the distribution of TMS-induced electrical effects with the individual connectivity distribution. These coil positions, along with standard scalp-measure-based coil positions, are entered into the neuronavigation system, and patients are randomised 1:1 for the targeting used to receive intermittent theta burst stimulation over 20-25 sessions. Patients and outcome assessors remain blind to the targeting method, with the primary outcome being the change in the Montgomery-Åsberg Depression Rating Scale from before to after treatment.

**Results:**

Interim analysis conducted by independent evaluators, based on results from the first 20 patients per group, suggested over 80% power to detect group differences at an alpha level of 0.048, with 40 patients in each group.

**Conclusions:**

To our knowledge, these would be the first results from a randomised prospective trial indicating the superiority of functional targeting over standard scalp measure-based targeting. While these targeting methods require further refinement, clinical applications will depend on the balance between effectiveness and cost compared to standard targeting. Related studies exploring the relationship between targeted brain connections and clinical outcomes may illuminate the brain-level mechanisms underlying recovery from depression.

**Disclosure of Interest:**

None Declared

